# Aquaporins and Animal Gamete Cryopreservation: Advances and Future Challenges

**DOI:** 10.3390/ani12030359

**Published:** 2022-02-02

**Authors:** João C. Ribeiro, David F. Carrageta, Raquel L. Bernardino, Marco G. Alves, Pedro F. Oliveira

**Affiliations:** 1 Clinical and Experimental Endocrinology, UMIB—Unit for Multidisciplinary Research in Biomedicine, ICBAS—School of Medicine and Biomedical Sciences, University of Porto, Rua Jorge Viterbo Ferreira 228, 4050-313 Porto, Portugal; joaopcribeiro95@gmail.com (J.C.R.); davidcarrageta@gmail.com (D.F.C.); raquellbernardino@gmail.com (R.L.B.); alvesmarc@gmail.com (M.G.A.); 2 QOPNA & LAQV, Department of Chemistry, University of Aveiro, Campus Universitário de Santiago, 3810-193 Aveiro, Portugal

**Keywords:** aquaporin, assisted reproductive technology, controlled-rate slow freezing, cryobiology, cryopreservation, cryoprotectant, embryo, oocyte, osmoregulation, spermatozoa, vitrification

## Abstract

**Simple Summary:**

Cryopreservation is the method for the long-term preservation of gametes and embryos. In recent years, intensive research has focused on improving cryopreservation protocols for the determination of optimal freezing conditions and cryoprotective agents’ concentration for each cell type. The optimal cryopreservation protocol comprises the adequate balance between the freezing rate and the correct concentration of cryoprotective agents to achieve controlled cellular dehydration and minimal intracellular ice formation. Osmoregulation is, therefore, central in cryobiology. Water and some solutes can cross the plasma membrane, whereas facilitating transport takes a great part in intracellular/extracellular fluid homeostasis. Cells express water channels known as aquaporins that facilitate the transport of water and small uncharged solutes on their plasma membrane, including some cryoprotective agents. This review explores the expression and the function of aquaporins in gametes and embryos. In addition, the putative role of aquaporins for cryopreservation procedures is discussed.

**Abstract:**

Cryopreservation is globally used as a method for long-term preservation, although freeze-thawing procedures may strongly impair the gamete function. The correct cryopreservation procedure is characterized by the balance between freezing rate and cryoprotective agents (CPAs), which minimizes cellular dehydration and intracellular ice formation. For this purpose, osmoregulation is a central process in cryopreservation. During cryopreservation, water and small solutes, including penetrating cryoprotective agents, cross the plasma membrane. Aquaporins (AQPs) constitute a family of channel proteins responsible for the transport of water, small solutes, and certain gases across biological membranes. Thirteen homologs of AQPs (AQP0-12) have been described. AQPs are widely distributed throughout the male and female reproductive systems, including the sperm and oocyte membrane. The composition of the male and female gamete membrane is of special interest for assisted reproductive techniques (ART), including cryopreservation. In this review, we detail the mechanisms involved in gamete cryopreservation, including the most used techniques and CPAs. In addition, the expression and function of AQPs in the male and female gametes are explored, highlighting the potential protective role of AQPs against damage induced during cryopreservation.

## 1. Introduction

Cryopreservation is used as an efficient method for the long-term storage of reproductive cells and embryos to use in human and animal-assisted reproduction [1,2]. In animals, gamete cryopreservation has great relevance to genetic selection and disease control. With cryopreservation techniques, it is possible to select the most suitable and commercially valuable gametes of animals and maintain desired genetic features. Moreover, long-term storage facilitates gametes transport over distances [3]. The first protocols were developed a long time ago, but the principles applied are similar and some remain fairly useful. The first report concerning cryopreservation is authored by Lazzaro Spallanzani, who cryopreserved sperm that regained motility upon thawing, as early as 1776. Remarkably, Spallanzani used snow as the freezing agent [4]. In the last century, assisted reproductive techniques (ART) and, in particular, cryopreservation techniques have drawn an intense focus of research. Christopher Polge et al. were successful in cryopreserving sperm and accidentally discovered the beneficial properties of glycerol as a cryoprotective agent (CPA) in 1949 [5]. In 1952, Polge and Rowson performed the first successful artificial insemination from cryopreserved bull sperm [6]. In the next year, Bunge and Sherman achieved successful pregnancies and live births with cryopreserved human sperm [7]. Ten years later, Sherman reported that cryopreserved human sperm had no loss of motility after one year of storage [8]. In the same year, Perloff described a protocol for the storage of human spermatozoa for more than five months without loss of viability [9]. Since then, cryopreservation techniques were rapidly improved to achieve increased success rates.

Notwithstanding all the improvements in the gamete cryopreservation technique over the years, this process causes damage to the cell and compromises cellular functions and the ability for fertilization. Gametes are subjected to mechanical, chemical, osmotic, and thermal stresses during the freezing and thawing stages [10,11,12]. A high number of factors can affect the efficiency of gametes’ cryopreservation, namely, the species of the gametes and embryos, the composition of the cryopreservation medium, temperature of incubation, and method of addition and dilution of CPAs. In addition, cellular features such as plasma membrane composition and osmoregulatory capacity are central to the success of cryopreservation methods [13]. Osmoregulation is, therefore, central for successful cryopreservation procedures. Water and some solutes can cross the plasma membrane, whereas facilitating transport takes a great part in intracellular/extracellular fluid homeostasis. Cells express water channels that facilitate the transport of water and small uncharged solutes on their plasma membrane, including some cryoprotective agents, the aquaporins (AQPs). Based on the current literature, we review the mechanisms involved in gamete cryopreservation of animals, detailing the most used techniques and CPAs. In addition, the expression and function of AQPs in the male and female gametes are explored, highlighting the potential protective role of AQPs against damage induced during cryopreservation.

## 2. Cryopreservation Techniques of Gametes and Embryos

Cryopreservation techniques use very low temperatures for the preservation of gametes and embryos within specific media by drastically reducing chemical kinetic reactions [14]. This approach represents an option for fertility preservation of germs cells for later use. The preservation of cells at very low temperatures, however, is very challenging due to the onset of cryoinjury. On the following topics, the use of CPAs, their characteristics, and their importance for successful cryopreservation techniques and prevention of cryoinjury will be discussed. Additionally, the most well-known and used cryopreservation methods, controlled-rate slow freezing and vitrification, will be detailed, including the advantages and drawbacks of each technique.

### 2.1. Cryoinjury

Water solidification into ice consists of the formation of a structured crystal at low temperatures, as six water molecules interact through hydrogen bonding [15,16]. Water–ice transition involves alterations in water’s ability to solvate salts and other solutes. Solutes are excluded from ice crystal structures resulting in hyperosmotic conditions. In cellular cryopreservation, cells are excluded from the forming ice matrix and the increased solute concentration constitutes a major damaging factor during this process. Gamete cryopreservation leads to several cellular and molecular alterations (reviewed in detail by Estudillo et al. [17]). Among them, the most important to highlight are the damage to the cellular and intracellular membranes [18] and ROS formation [19]. As a consequence of very slow freezing rates, an efflux of intracellular water occurs, leading to an intracellular hyperosmotic environment that prevents the formation of ice [20,21]. Cellular dehydration can have deleterious effects including irreversible membrane damage and cell lysis on thawing [22]. On the other hand, very high cooling rates also lead to cellular damage due to the formation of intracellular ice [23]. In addition, increased ROS formation occurs due to mitochondria malfunction by electron leakage [24]. Mitochondrial malfunction, however, can also be related to mitochondrial membrane integrity [25]. Consequently, excessive ROS formation can be the starting point to other damage such as increased lipid peroxidation, protein oxidation via carbonylation, and DNA damage [26,27,28].

Cells can withstand cryopreservation processes if an optimal cooling rate is determined and if the proper CPA is added to the cryopreservation medium. The optimal cryopreservation protocol, therefore, comprises the adequate balance between freezing rate and the correct concentration of CPAs to achieve cellular dehydration and minimal intracellular ice formation. The correct concentration of CPAs, in turn, reduces the osmotic shock and simultaneously avoids cytotoxic effects.

### 2.2. Cryoprotective Agents (CPAs)

CPA are, by definition, any solute that allows higher post-thaw recovery rates when added to the cryopreservation medium [29]. In other words, CPAs are chemical compounds used for cryoinjury prevention during cryopreservation techniques that can modulate the interaction between water molecules and their interaction with other solutes as ice nucleation occurs [30]. In the intracellular environment, CPAs replace water and hamper the hydrogen bonding between water molecules, therefore reducing the formation of intracellular ice. In addition, CPAs also reduce the accumulation of solutes and prevent hyperosmotic conditions due to the increased hydration status of the cell. On the other hand, very high concentrations of penetrating CPAs (molar range) result in cellular toxicity or even apoptosis due to osmotic stress or osmotic shock [31]. For cryobiology scientists, the use of CPAs has become a routine, even though a clear understanding of their mechanisms of action and long-term consequences are, to some extent, still unknown. It is important to highlight that different cell types or even the same cell type from different species require specific and optimal concentrations of CPAs for preventing cryoinjury and achieving successful cryopreservation. The data on protocols and selection of the most suitable CPAs for each cell type is steadily improving, as intensive research is conducted for the detection of hidden cryopreservation-induced damage which sometimes takes a longer period to develop [31]. Curiously, CPAs research lean towards empirical observations and trial-and-error methods. As aforementioned, one of the first identified CPAs was glycerol, in a fortuitous identification due to the inadvertent use of a mislabeled bottle containing a glycerol mixture [5].

CPAs are generally classified as penetrating or nonpenetrating, according to their plasma membrane permeability [23]. Penetrating CPAs are commonly organic compounds characterized by a low molecular weight (generally less than 100 Dalton) and high amphiphilic properties, which confers permeability through the plasma membrane [32]. The most well-known and used penetrating CPAs are glycerol, ethylene glycol, propylene glycol (1,2-propanediol), dimethyl sulfoxide (DMSO), methanol, and butanediol (characteristics and toxicity are extensively reviewed by Elliott et al. [31]). DMSO and glycerol, two very well-known and widely used penetrating CPA, are reported to interact with head groups of phospholipids within the lipid bilayer, conferring stability [33,34]. DMSO is reported to decrease the membrane conformational disorder conferred by freezing into low temperatures (at least −40 °C) [35]. Glycerol interacts with membrane phospholipids reducing the freezing-induced damage and facilitating the water efflux from the cells during temperature decrease and consequent dehydration [36,37]. Membrane and intracellular organelle stability are essential for gamete cryopreservation. Mammalian oocytes, for instance, usually suffer destabilization of the meiotic spindle, a tubulin-based network that controls chromosomal distribution, during cryopreservation procedures [38,39]. Johnson and Pickering reported that the presence of DMSO in the cryopreservation media was found to stabilize microtubule structure in mouse oocytes. If the exposition is prolonged, however, spindle destabilization was reported to occur as a consequence of DMSO presence [40]. Van der Elst et al. reported that both propanediol and DMSO stabilize spindles of mouse oocytes at high concentrations (1.5 M) [41]. Washing out propanediol and returning to physiological temperatures, however, led to spindle destabilization, which did not occur when DMSO was used. In a later study, Gook et al. reported that propanediol at high concentrations (1.5 M) had a protective effect in human oocytes [42]. In support of the results obtained by Van der Elst et al., Gook and collaborators did not observe protective effects of propanediol in mouse oocytes, highlighting the necessity of specific protocols and the use of specific CPAs in cryopreservation media to cover species-dependent differences of gametes.

Nonpenetrating CPAs are high molecular weight compounds that are usually highly hydrophilic. Nonpenetrating CPAs remain in the cryopreservation medium, protecting cells from high osmotic stresses and conferring less toxicity [23]. The most common nonpenetration CPAs are polyvinylpyrrolidone (PVP), polyethylene glycol, and sugars such as sucrose and trehalose [43,44]. Interestingly, a combination of penetrating and nonpenetrating CPAs is usually applied for reducing cryopreservation-induced damage in cells [45,46,47]. As aforementioned, DMSO, a very well-known and widely used penetrating CPA, is reported to interact with head groups of phospholipids within the lipid bilayer, conferring stability. At higher concentrations (>7 mol %), however, DMSO causes the thinning of lipid bilayers, leading to membrane damage and cytotoxicity [48]. On the other hand, if nonpenetrating CPAs are combined, such as sugars or polymers, there is the formation of hydrogen bonds with the lipids present in the plasma membrane, reducing the deleterious effects of DMSO [33,49]. In vitrification protocols, penetrating CPAs are used at even higher concentrations. Hotamisligil et al. reported that exposure to propanediol at very high concentrations (6 M) for short periods (3–5 min) did not affect microfilament stability in mouse oocytes, whereas longer exposition times (7–10 min) resulted in disruption [50]. It is important to highlight that CPAs are usually applied in the molar concentration range. Such high concentrations have deleterious effects on cells and may affect enzymes, transporter mechanisms, ion exchange, metabolic processes, or even gene expression. Unsurprisingly, these adverse effects are heavily linked to permeating CPAs, whereas nonpermeating CPAs have little evidence of cytotoxicity. Besides, the accumulation of penetrating CPAs within cellular compartments is an issue. After thawing, washing out CPAs with isotonic solutions results in water influx into the cell cytoplasm, leading to cellular damage and loss of membrane integrity [31]. An applied solution is the gradual dilution of CPAs in slightly less hypertonic media [51,52]. Nonpenetrating CPAs (often at similar or slightly lower concentrations than the penetrating CPAs) are used to assist in water influx upon thawing. Sugars, such as sucrose, have been widely studied due to their cryoprotective effects and mitigation of water influx during thawing procedures [53,54,55]. Sucrose is widely used as a CPA in cryopreservation media for human oocytes. Oocytes have a particularly distinct membrane composition and are sensitive to transmembrane water fluxes during thawing [56]. Several studies highlight that the addition of sucrose (0.2–0.3 M) improves post-thawing recoveries and higher live births rates following cryopreservation [57,58,59]. Taken together, the complex interaction between high CPA concentration, freezing rate, and time of exposure makes the optimization of cryopreservation protocols very challenging.

### 2.3. Controlled-Rate Slow Freezing

**Figure 1:**
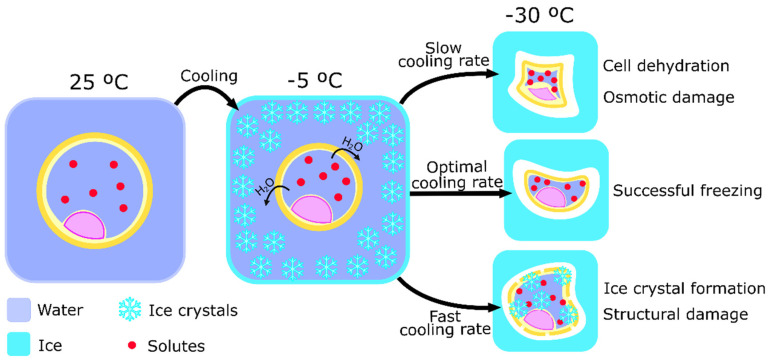
Schematic representation of the process of controlled-rated slow freezing and the importance of cooling rate to cell cryopreservation. Ice crystal formation starts around the cell while it loses water volume, after initial cooling to around −5 °C. The importance of the cooling rate is represented by its relative value to the optimal cooling rate and the potential damages caused by the use of inappropriate cooling rates. A low cooling rate will cause cell dehydration and osmotic damage by solute concentration, causing chemical damage. On the other hand, a high cooling rate increases the probability of ice crystals formation inside of the cell, causing structural damage.

Controlled-rate slow freezing was the first technique to be developed that allowed the cryopreservation of gametes and embryos. In 1971, Whittingham was able to describe a protocol that cryopreserved mouse embryos at a rate of approximately -1 °C per second [60]. Throughout the years the protocol suffered modifications and, currently, still is widely used for embryo cryopreservation [61]. Nowadays, cells are usually diluted in medium solutions with moderate CPAs concentration (up to 1.5 M). Then, the distribution of cells in small volumes within thin straws facilitates temperature balance throughout the sample. After cooling to a raging temperature of −5 to −7 °C, cells are exposed to a slow temperature decrease of about 0.3 to 0.5 °C per minute until reaching a temperature between −30 and −65 °C. Straws are then directly immersed into liquid nitrogen to rest for as long as cryopreservation is needed [62]. This procedure is unique by its slow cooling rate that, in combination with the adequate concentration of CPAs, ensures that the formation of ice crystals only happens outside the cell [17]. This mechanism happens due to slow and controlled loss of water as the temperature decreases and consequent concentration of the solutes that prevent ice crystals formation in the intracellular medium. On the other side of the spectrum, excessively slow cooling will dehydrate the cell cytoplasm, creating excessive solute concentrations that could lead to cell death after long-term exposure to such conditions [63]. The optimal cooling procedure, therefore, is a balancing act between preventing the formation of ice crystals and the toxicity of high solute concentrations inside of the cell (Figure 1).

### 2.4. Vitrification

**Figure 2:**
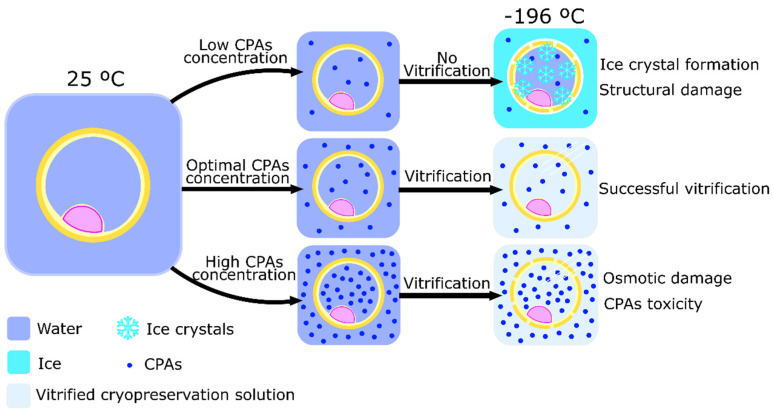
Schematic representation of the process of vitrification and the importance of cryoprotectants agents (CPAs) to achieve cell cryopreservation. The use of different CPAs concentrations causes different cell damages after the vitrification process. Low CPAs concentration does not allow vitrification and allows ice crystals inside of the cell, causing structural damage. High CPAs concentration increases medium toxicity and osmotic damage, inflicting chemical damage to the cell.

Vitrification was developed to simplify the process of cryopreservation through the controlled-rate slow freezing method. Vitrification was first utilized to cryopreserve tissues [64]. The essence of this method is to completely overpass ice crystal formation. To do so, two different approaches can be taken. The first protocol developed to cryopreserve embryos by vitrification was used by Rall et al. in 1987 [65]. This protocol is based on the incubation of embryos and oocytes in a solution of multiple CPAs in high concentrations (up to 8 M) [17,63]. Then, embryos or oocytes are divided into thin straws and submitted to cooling rates of −200 °C per minute. Usually, the straws are dropped directly into liquid nitrogen (−196 °C) where they can be rapidly cooled and vitrified. Then, samples can be maintained in liquid nitrogen to rest. The high concentration of CPAs has two essential purposes: increase the viscosity of the medium to dehydrate the cells, and avoid water crystallization [66]. This technique is, therefore, a balancing act between the toxicity of the CPA and the formation of ice crystals throughout the media (Figure 2). The former is the reason this approach is more widely used in oocytes and embryos, but not so much in spermatozoa. Spermatozoa are more susceptive to the toxicity caused by high concentrations of CPAs due to the higher sensibility to osmotic stress when compared to embryos and oocytes [67]. It is possible, however, to decrease the CPA concentration by 50 to 75% by using small volumes (1 to 1.5 µL) of vitrifying solution. The immediate heat dispersion after contact between the liquid nitrogen and the small sample volume enhance uniform vitrification, maintaining the liquid molecular structure despite being in a solid state, and thus, preventing ice crystal formation [66].

Recently, newer approaches to the vitrification process allow the application of this technique in the cryopreservation of sperm cells [68]. To do so, the investigators tried to decrease sample volume to facilitate heat diffusion and overtake low medium viscosity. The protocol developed is performed by incubating sperm cells in a solution of sucrose (0.5 M) for mild sperm cells shrinkage and then the direct release of small drops of the sperm solution directly into liquid nitrogen (−196 °C). This protocol has shown efficiency in cryopreserving sperm cells by preventing ice crystals formation, osmotic stress, and toxicity caused by a high concentration of CPAs [68]. Although this technique exhibits a high potential, it is, however, yet to be regarded as a routine sperm cryopreservation protocol [69]. On the other hand, the applicability of such a small concentration of CPAs in oocyte and embryo cryopreservation seems improbable due to their lower surface/volume ratio when compared to sperm cells, which is correlated with a higher probability of ice crystal formation. Nonetheless, oocyte and embryo have been cryopreserved using vitrification with a high concentration of CPAs for decades with increasing efficiency [62].

### 2.5. Controlled-Rated Slow Freezing Versus Vitrification: Advantages and Drawbacks

Controlled-rate slow freezing and vitrification were previously used to cryopreserve embryos of multiple mammal species. However, both techniques have different applicability to embryos of different species. It is hypothesized that this difference in response is due to variations on embryo membrane permeability and lipid content as well as embryo size, which causes the need for protocol optimization (cooling rates, CPAs, cryopreservation medium) between species. The first study that compared these two techniques was developed by Wurth et al. in 1993 [70]. These authors used bovine embryos to study the efficiency of the techniques in successful pregnancies. While 23% of the vitrified embryos resulted in pregnancy, only 14% of controlled-rate freezing embryos developed into a fetus [70]. The following study also performed with bovine embryos showed a similar result [71]. Recent studies that compared these two cryopreservation techniques, however, were not able to encounter significant differences between pregnancy rates of bovine embryos. Sanches et al. [72], in 2016, and Gómez et al. [61], in 2020, reported that the pregnancy rates of controlled-rate freezing embryos (40.2% and 59.7%, respectively) were statistically similar to the pregnancy rates of vitrified embryos (35.9% and 53.6%, respectively). It is, however, worth mentioning that the pregnancy rates have been increasing by either using controlled-rate slow freezing (from 14% in 1993 to 59.7% in 2020) or vitrification (from 23% in 1993 to 53.6% in 2020) since the first reports of bovine in vitro-derived embryo cryopreservation [61,70].

Caprine embryos have been shown to present better viability after the vitrification process than after controlled-rate slow freezing [73,74,75]. On the other hand, studies performed in equine and donkey embryos showed that controlled-rate slow freezing seemed to be the most appropriate cryopreservation method [76,77]. Studies on other animal species such as rabbit and cat embryos did not present any difference in survival from the processes of vitrification or controlled-rate slow freezing [78,79]. The literature on other animal species embryo cryopreservation presents contradictory results. In 1999, a study by Uechi et al. described that significantly lower numbers of vitrificated two-cell mouse embryos were successfully developed when compared to the percentage of the embryos cryopreserved by controlled-rate slow freezing [80]. Two following studies, however, showed that vitrification of mouse embryos and blastocysts resulted in higher survivor rates when cryopreserved by controlled-rate slow freezing [81,82].

In avian species, the embryo from Japanese quail presented better outcomes after a vitrification cycle than after controlled-rate slow freezing [83]. In fish, namely zebrafish, embryo cryopreservation protocols are yet to be developed [84]. This could be due to different membrane permeability properties due to adaptation to the fact that fecundation happens underwater [85,86]. To tackle this problem, studies already investigated the effect of both cryopreservation procedures in whole ovarian tissue and testis of zebrafish [84,87]. Zebrafish oocyte’s cryopreservation presented better results after vitrification of ovarian tissue [87].

Embryo and oocytes are challenging to cryopreserve. The high volume of the cells as well as plasmatic membranes with low permeability difficult the process of cryopreservation [62]. Moreover, the different results described between species do not allow to choose of a standard cryopreservation method. Some studies even show different results for the same animal models, which can be explained by the usage of different protocols (cooling rates, CPAs, and cryopreservation medium). In general, the literature indicates that the vitrification protocol works better for oocytes and embryos since these cells are larger and less permeable when compared to sperm cells. Membrane permeability is crucial for preventing intracellular ice crystal formation during the controlled freezing protocol. Despite that, both cryopreservation protocols inflict damage and changes to the cells on multiple fronts (for review see [17]). Controlled-rate slow freezing was found to inflict more structural damage to mitochondria and other organelles, including cytoskeleton, plasmatic and nuclear membrane in bovine embryos [88]. In mouse oocyte and blastocyst, the controlled-rate slow freezing technique has been shown to increase DNA fragmentation and reactive oxygen species (ROS) production when compared to vitrification [89,90]. On the other hand, vitrification increased gene expression changes in sheep blastocyst when compared to the ones noted in the controlled-rate slow freezing method [91]. Mouse vitrified embryos, as well as the resulting blastocysts, presented less glucose consumption when compared to controlled-rate slow freezing embryos and resultant blastocysts [80]. Thus, damage caused by the controlled-rate slow freezing method is attributed to mechanical injuries caused by ice formation. On the other hand, the vitrification method seems to impair cellular metabolic profile and gene expression without mechanical injuries. Hence, more studies are needed to understand the possible underlying damages caused by vitrification.

Sperm cells are generally more resistant, thus, respond better to cryopreservation. Most of the literature comparing both methods of cryopreservation shows that sperm cells respond better to controlled-rated slow freezing than to vitrification, contrasting to oocytes and embryos. Vitrification decreases sperm parameters in sperm of mammalians such as mouflon, donkey, buck, monkey, and stallion in a much higher amplitude than controlled-rate slow freezing [92,93,94,95,96]. Similar results are also seen in avian species. Spermatozoa from cockerels presented better viability and motility after controlled-rate slow freeze than vitrification [97]. Opposite to oocytes and embryos, sperm cells have highly permeable plasmatic membranes as well as small volume which prevents the formation of ice crystals during controlled-rate slow freezing cryopreservation [98]. Moreover, the use of high concentrations of CPAs characteristic of the vitrification process is toxic for sperm cells. This could be the cause of the low percentages of motility in vitrified sperm cells from the mentioned studies (22.4 to 1.2%) as compared to the resultant of controlled-rate slow freezing (42 to 49.4%) [94,95,96]. Further studies are still needed to improve vitrification protocols with lower concentrations of CPAs, which could overtake a drawback of the technique and lead to compelling results.

Osmotic homeostasis is, therefore, central in cryobiology. Water and some solutes can cross the plasma membrane, however, facilitating transport takes a great part in intracellular/extracellular fluid homeostasis. Cells express water channels that facilitate the transport of water and small uncharged solutes on their plasma membrane, including some CPAs, the aquaporins (AQPs). The expression, function, and putative role of the different AQPs homologs during cryopreservation protocols will be detailed in the following topics.

## 3. Aquaporins (AQPs) and Cryotolerance: Expression and Function in Spermatozoa, Oocyte, and Embryos

The osmoregulation includes the movement of water and other small molecules across cell membranes, which are essential in many of the biological processes including the natural process of reproduction of species and gametes’ cryopreservation. AQPs are the principal channel proteins related to fluid movements in biological membranes [99]. AQPs are a family of channels permeating to water, small solutes, and gases across biological membranes. There are 13 homologs (AQP0-12) identified in mammals, which are grouped based on their biophysical properties. The orthodox AQPs are primarily permeable to water (AQP0, AQP1, AQP2, AQP4, AQP5, AQP6, and AQP8). The aquaglyceroporins (AQP3, AQP7, AQP9, AQP10) transport a series of small uncharged solutes, particularly glycerol, in addition to water [100]. AQP11 and AQP12 are considered unorthodox aquaporins, due to conducting properties being unclear [101]. AQP8, due to its marked ability to transport ammonia and hydrogen peroxide, besides water, is also indicated as ammoniaporin or peroxiporin [102]. The biological significance and translational role of AQPs have been the object of intense investigation in the male and female reproductive tract [103], including their role in osmoregulation, gamete physiology, and cryopreservation [104,105]. In the next topics, it will be discussed the current literature on AQP expression in the membranes of germ cells and embryos of animals (Table 1). It also will be given the focus on the role of the AQPs in the function of these cell types.

### 3.1. AQPs Expression and Function in Spermatozoa

Sperm plasmatic membranes were found to express AQP1, AQP3, AQP7, AQP8, AQP9, AQP10, and AQP11. The number of AQPs expressed and their localization, however, is species-dependent and, in some cases, even individual-dependent. In mammals, only canine sperm was reported to express AQP1 [106]. Canine sperm presents low water permeability and AQP1 is especially permeable to water [133]. The authors, however, highlight that the expression level of this AQP was very small, not allowing its detection on immunocytochemistry assays [106]. AQP1 paralogs, AQP1ab and AQP1aa, are also found on sperm cells of marine teleost seabream [85,108]. While AQP1aa is expressed in the tail, AQP1ab is expressed in the head. Interestingly, AQP1ab expression increased in the sperm’ head of marine teleost fishes after sperm motility activation. The authors hypothesized that this increase in AQP1ab expression was related to more intracellular vesicles that could be necessary for motility maintenance; however, more studies are needed to clarify the role of this AQP [85,108]. AQP1aa was pointed to have a role in water efflux to respond to hyperosmotic shock on sperm from marine teleost [108,134].

AQP3 expression is encountered in the midpiece of multiple mammals’ species including pig, mouse, and bull [105,110,111]. Moreover, it is also present in the tail of mouse and pig sperm and the acrosomal region of pig sperm [105,110]. Stallion sperm cells also express AQP3; however, no specific localization was reported [122]. In functional studies, AQP3 was pointed as an osmotic sensor specialized in the efflux of water important for the osmoadaptation to the osmolarity of the female reproductive tract fluid [110]. However, AQP3 expression on pig acrosomal region could highlight a role in acrosomal reaction [105].

AQP7 is present in the midpiece of rat and bull spermatozoa [118,119] and in the tail of rat, mouse, pig, and bull sperm cells [118,119,120,121]. Bull sperm cells also express AQP7 in the plasma membrane of the acrosome [119]. Stallion sperm cells express AQP7, but, like AQP3, no specific localization has been reported [122]. In avian species, geese spermatozoa express AQP7 in the tail section [123]. Moreover, the seabream sperm cell head also expresses AQP7 [85], thus making this AQP expressed also in marine sperm. Despite the wide expression throughout the different animal species, AQP7 knockout studies in mice did not show any effect on animal fertility [124,135]. One study points to the involvement of AQP7 in the sperm cells permeability to solutes instead of water like AQP3 [124]. The characteristic diffuse expression of AQP7 throughout the sperm cells body (in most mammalian species) could indicate a role as the main solute channel. Solutes such as glycerol can be used as an energy source for sperm cells [136] but also to regulate sperm volume in hypertonic solutions. It is also worth mentioning the role of AQP7 on the spermatogenic process of rat and seabream sperm cells [85,137]

AQP8 is an orthodox aquaporin that is also found in mice and dog spermatozoa and rat spermatogenic cells [124,125,138]. However, its localization is not yet specified. By being an orthodox AQP and thus, highly permeable to water, studies indicate that AQP8 in mice spermatozoa have a major role in water mobilization for sperm volume regulation [124,139]. In fish spermatozoa, teleost seabream expresses a AQP8 paralog: AQP8b [127]. This AQP was found to be important in the management of hydrogen peroxide formed in the sperm mitochondria, allowing its proper functioning [127,140].

AQP9 was only confirmed on pig spermatozoa in the acrosomal membrane [121]. It is interesting the expression of a unique AQP on pig sperm cells since most mammal sperm have AQP7 in the acrosomal area [119,141]. More studies are needed to clarify the role of AQP9 in pig sperm. Other uniquely expressed AQP is the AQP10 paralog AQP10b in teleost seabream sperm [85]. There, AQP10b was found to be expressed throughout the body of the sperm. This protein was found to translocate into the sperm membrane after motility activation [129]. That phenomenon can be explained by the extra permeability needed in sperm of sea animals after motility activation.

AQP11 is also widely expressed throughout mammalian species. It can be encountered in the head, midpiece, and diffusely in the tail of pig spermatozoa [130] as well as in stallion sperm [122]. Bull sperm also present AQP11 in multiple sections, namely, head and throughout the tail [104]. In rats, this AQP is present in the distal part of the tail sperm [131]. AQP11 is a superaquaporin, and thus, is regarded as an intracellular AQP [142]. While that may be true to the expression seen in sperm’s head, making this AQP the one responsible for the membrane permeability of the organelles there present [141], the same cannot be said to the expression noted in the sperm tail where there are no known organelles. A function for this expression patter is still to be elucidated. Some studies show that AQP11, like AQP7, is important in the process of water volume reduction during spermatogenesis [131]. Despite that, AQP11 expression was positively correlated with membrane fluidity and sperm motility in pigs [130].

As demonstrated by the studies herein presented, the overlap of the expression of AQPs in sperm cells is clear. Spermatozoa, however, are highly compartmentalized cells which may emphasize the importance of individual membrane permeabilities. The presence of two AQPs with the same function and permeability is rare. Nevertheless, it is still important to gather knowledge on AQP expression and function to better understand sperm function and optimize cryopreservation techniques.

#### AQPs expression and Cryotolerance in Spermatozoa

In pig sperm cells, AQP3 and AQP7 (but not AQP11) expression levels were positively correlated with the ability of sperm cells to survive a cryopreservation cycle [105]. Contrastingly to pig sperm, bull sperm AQP11 expression levels showed to be higher in sperm that respond better to cryopreservation than the ones seen in sperm cells that responded poorer to cryopreservation [104]. Another report on bull sperm, however, stated that AQP3 and AQP7 were particularly important for the maintenance of sperm motility and velocity after a cryopreservation cycle [143]. Moreover, stallion sperm cryotolerance was found to be related to the expression of AQP3 and AQP11, but not AQP7. Taking this into consideration, it is hypothesized that the cryotolerance of different species of sperm is dependent on the expression of different AQPs. It is worth noticing, however, that all the AQP mentioned so far are all aquaglyceroporins (except for AQP11, which also transport glycerol) [103]. Studies in stallion and bull sperm confirm that orthodox AQPs do not affect ejaculate cryotolerance, however, aquaglyceroporins inhibition decreased sample cryotolerance [144,145]. The inhibition of aquaglyceroporins with phloretin decreased the cryotolerance of samples with proven good cryotolerance but not of samples with bad cryotolerance [146]. This fact highlights that aquaglyceroporins have a crucial role in cryopreservation survival and damage control. These proteins allow the sperm cells membrane to survive the osmotic shock after cryopreservation medium incubation and also during the thawing process, after isotonic medium dilution, preserving sperm membranes integrity, including the mitochondrial membrane [145]. That could indicate that aquaglyceroporins can also mitigate the formation of excessive ROS and prevent oxidative stress in sperm intracellular space. On the other hand, the peroxiporin AQP8 plays a role in hydrogen peroxide diffusion. Inhibition of AQP8b in seabream sperm resulted in ROS accumulation in the mitochondria matrix that resulted in mitochondrial membrane depolarization and consequently diminished sperm motility [127]. This could indicate a potential role of AQP8 in ROS diffusion (namely, hydrogen peroxide) after cryopreservation. AQP8 is also present in the mitochondrial membrane of human sperm [141]; however, it was not found in rat sperm [147]. Thus, further studies are needed to elucidate the role and expression of AQP8 in mammalian sperm mitochondrial membrane and its possible relation with cryotolerance.

### 3.2. AQPs Expression and Function in Oocytes and Embryos

The expression and function of AQPs in mammalian oocytes are poorly characterized. Few studies focused on the expression of AQPs in oocytes from different species and their role besides the putative water and ionic regulation remains to be elucidated. Edashige et al. were pioneers in this field and studied the expression of *Aqp1–9* mRNA in ICR mice oocytes, where only the expression of *Aqp3* and *Aqp7* mRNA were detected [112]. These authors observed a low expression of both homologs, suggesting that the low contribution of AQPs for water transport would explain the observable low permeability parameters of mice oocytes [113]. In another study using oocytes from ICR mice, Woo Jo et al. observed that the *Aqp3* mRNA expression was higher in immature than on mature oocytes, suggesting a role during oocyte maturation [114]. In a later study, Ya-Jing et al. identified the expression of AQP9, besides AQP3 and AQP7, in oocytes from C57BL/6J mice [115]. In addition, these authors also observed translocation of AQP7 but not of AQP3 and AQP9 into the plasma membrane following osmotic stress, which highlights the importance of AQP7 for fluid and ionic homeostasis in oocytes from this species. Ford et al. studied the expression of *Aqp1-9* mRNA and only identified *Aqp9* mRNA in immature oocytes from Wistar rats [128]. The expression of *Aqp9* mRNA, however, was not found in mature oocytes, whose permeability was not affected by phloretin, a general AQP inhibitor, and remained unaltered during mannitol hypo-osmotic swelling assays.

The study of AQPs in oocytes from teleost fishes produced compelling results. Teleosts are constantly exposed to different osmotic gradients due to their underwater inhabitation. As a result, the osmoregulatory capacity of these organisms is high [148]. The oocytes and embryos from marine oviparous teleosts, however, do not possess the same osmoregulatory capacity. Oocytes from marine teleosts hydrate during oocyte maturation, before ovulation, as an adaptation to support the hyperosmotic conditions found at seawater. The hydration process confers a water reservoir in oocytes, which compensates for the passive water efflux due to the hyperosmotic environment until the development of osmoregulatory organs [149]. These species express AQP1ab, an AQP1 paralog, which is responsible for the water transport that occurs during the hydration of the oocytes [109]. The expression of AQP1ab has been identified in several species although species-specific localization in the oocyte has been reported [150,151,152,153]. Compelling evidence demonstrated that the swelling of oocytes during hydration is inhibited by mercury, a known inhibitor of AQPs [153,154,155]. The expression of AQP1ab has also been identified in some freshwater species, suggesting some hydration of their oocytes [156,157]. The mechanisms and extent of hydration in oocytes from freshwater species, however, remains unknown.

The blastocyst is formed when the oocyte is fertilized and undergoes a cleavage stage characterized by several mitotic divisions, which are then capable of initiating uterine implantation. During embryo development, a high rate and differential gene expression occur [158]. In the aforementioned study by Edashige et al., it was reported that *Aqp3* and *Aqp7* mRNA were expressed during all stages of ICR mice embryo development [112]. Interestingly, Xiong et al. observed that the knockout of either AQP3 or AQP7 significantly inhibited preimplantation embryo development in mice [116]. The expression of other AQPs homologs, however, varies during different stages of embryo development. Edashige et al. also identified *Aqp8* and *Aqp9* mRNA in ICR mice blastocysts [112]. The expression of *Aqp8* mRNA was also found in the 4-cell stage of mice embryos, which was further increased during the 8-cell stage [126]. Barcroft et al. reported the expression of AQP9 in 8-cell stage embryos of CD-1 × CB6F1/J mice [117]. In addition, AQP9 was also found expressed in the apical membrane of the blastocyst’s trophectoderm, whereas AQP3 and AQP8 were found expressed in the basolateral membrane. In another study by the same group, it was reported that *Aqp1* mRNA was expressed in the 2-, 4-, 8-cell stage embryo, and blastocyst, whereas *Aqp5* and *Aqp6* mRNA were identified in all stages of murine preimplantation development [107]. In a later study, Offenberg and Thomsen reported that also *Aqp11* but not *Aqp12* mRNA is expressed during all stages of murine preimplantation development [132]. Taken together, the expression of AQPs in the preimplantation stages suggests that AQPs may play a role during cavitation [117].

The most expressed AQP homologs in both oocytes and embryos are aquaglyceroporins. Aquaglyceroporins mediate the transport of both water and glycerol, which highlights the potential role of glycerol for oocyte and embryo development. Differences between species or even between different strains, however, remain to be disclosed [98]. In addition, little is known concerning the expression of AQPs in oocytes from non-rodent species. Nevertheless, these findings highlight that the presence of AQPs is essential for the maintenance of osmotic equilibrium in oocytes and embryos.

#### AQPs Expression and Cryotolerance in Oocytes and Embryos

As compared to sperm, oocytes express fewer AQPs homologs. Likewise, studies concerning AQP expression and function, including studies focusing on cryopreservation, in oocytes are scarce in comparison to studies focusing on sperm. Oocytes are also far less permeable to water, which is considered to move through oocytes mainly by simple diffusion. Similar permeability parameters were observed in early-stage mouse embryos as compared to oocytes, but in morulae and blastocysts, in contrast, water moves through the plasma membrane by facilitated diffusion, mainly via AQP3 [159]. AQP3 is the most expressed AQP homolog in mammalian oocytes and embryos, thus the majority of studies focus on its expression and role. AQP3, therefore, seems to play a major role in the transport of water and small solutes, including CPAs, across oocytes and embryos’ plasma membranes. In mouse oocyte and early embryos, permeability to glycerol was described as low, whereas in morulae, the permeability to glycerol was found higher [160,161]. As aforementioned, AQP3 expression is increased in mouse morulae, which can explain the observed results. In support, permeability to glycerol significantly decreased in mouse morulae by suppressing the expression of AQP3 [161]. Similar results were found for ethylene glycol, which was hypothesized to be transported into the cell by AQP3. In contrast, DMSO permeability of mouse oocytes and early-stage embryos were low whereas the permeability of morulae was found to be higher, which were unaffected by the suppression of AQP3 expression. These results suggest that DMSO is transported by facilitated diffusion, thus via a channel other than AQP3 [161]. Similar results for glycerol and ethylene glycol permeability were reported in both bovine and pig oocytes, early-stage embryos, and morulae, but permeability to DMSO presented conflicting results in these cells [162,163].

Studies focusing on the artificial expression of AQP3 in oocytes reported compelling results. Edashige et al. studied whether artificial expression of AQP3 in mouse oocytes would improve membrane permeability to water and glycerol and oocyte survival after a cryopreservation procedure. After thawing, 74% of the overexpressing AQP3 oocytes survived whereas none of the oocytes from the control group survived. When oocytes were inseminated in vitro, the penetration rate was reported as 40% and the cleavage rate as 31%, highlighting that the overexpressing AQP3 oocytes kept their ability for fertilization [164]. In support of these results, Morató et al. induced the expression of human AQP3 and zebrafish Aqp3b-T85A mutant AQP3 paralog in porcine oocytes and observed an increased permeability to ethylene glycol. The zebrafish paralog was found to be more efficient than the human channel at increasing porcine oocytes permeability [165]. In a later study, Bedford-Guaus induced the expression of the zebrafish Aqp3b-T85A in porcine oocytes and not only reported an increased permeability to ethylene glycol but also higher post-thaw survival rates [166]. Valdez Jr. et al. had similar results when they induced the expression of rat AQP3 in immature medaka (*Oryzias latipes*) oocytes. In that study, it was reported that the overexpression of AQP3 also led to improved permeability to propylene glycol and ethylene glycol [167]. As a result of these studies, efforts have been made for the characterization of zebrafish AQP3 paralogs, which exhibit enhanced permeability to CPAs and its induced expression could improve cryopreservation outcomes [168].

Studies concerning AQPs expression and function in embryos including studies focusing on cryopreservation, to the best of our knowledge, were not performed. Further research is needed, although studying embryos is a difficult process due to technical and ethical concerns.

## 4. Conclusions and Future Perspectives

During the last decades, many advances in the gametes and embryos’ cryopreservation techniques and composition of CPAs have been observed, which has led to greater success rates in long-term preservation. Different cell types, or even the same cell type but different species, have different membrane permeabilities and, consequently, differences in cryotolerance. Taking into consideration the data presented in this review, it is possible to affirm that membrane permeability is an important indicator of cryotolerance. Membrane permeability is directly correlated with membrane fluidity [169], which, in turn, is directly correlated with cryotolerance [170].

Protocols have been refined and different CPAs selected to improve cryopreservation methods for sperm, oocytes, and embryos. Despite all the advances in science and new techniques for gamete cryopreservation, there remains a lot of failure due to the damage caused by cryopreservation procedures, namely the stress induced by the freezing and thawing. Most of the protocols are obtained due to empirical observations and trial-and-error methods. The permeability of plasma membrane to water and CPAs is essential during cryopreservation. The composition of the plasma membrane and how its characteristics affect water and solutes permeability of the different gametes and embryos, however, remains poorly characterized. Membrane channels such as AQPs are essential in gamete and embryo cryopreservation due to their important role in the transport of water and non-charged solutes. By studying the plasma membrane composition of gametes and embryos, characterizing the differences between cell types and between the same cell type from different species, the current cryopreservation protocols can be improved and novel, specific, and targeted protocols can be developed.

In conclusion, understanding the roles of AQPs in gametes and embryos’ plasma membranes is a crucial step towards investigating potential implications in the success of cryopreservation. Novel data concerning the role and modulation of AQPs in gamete biology, and their role in cryopreservation, will prompt the development of novel and more effective protocols to minimize the risks inherent to cryobiology.

## Figures and Tables

**Table 1:** Expression and localization of aquaporins (AQPs) in spermatozoa, oocytes, and embryos in different animal species.

AQP Isoform	Spermatozoa					
	Head	Midpiece	Tail	Oocytes	Embryos	References
AQP1	Dog *	Dog *	Dog *	-	Mouse ^#^	[106,107]
AQP1aa	-	-	Seabream	-	-	[85,108]
AQP1ab	Seabream	-	-	Teleost fishes	-	[85,108,109]
AQP3	Pig Stallion *	Pig	Mouse	Mouse	Mouse	[105,110,111,112,113,114,115,116,117]
		Mouse	Pig			
		Bull	Stallion *			
		Stallion*				
AQP5	-	-	-	-	Mouse ^#^	[107]
AQP6	-	-	-	-	Mouse ^#^	[107]
AQP7	Bull	Rat	Rat	Mouse	Mouse	[85,112,113,115,116,118,119,120,121,122,123]
			Mouse			
	Seabream	Bull	Pig			
			Bull			
	Stallion *	Stallion *	Geese			
			Stallion *			
AQP8	Mouse * Dog *	Mouse * Dog *	Mouse * Dog *	-	Mouse	[112,117,124,125,126]
AQP8b	Seabream	-	-	-	-	[127]
AQP9	Pig	-	-	Mouse Rat ^#^	Mouse	[112,115,117,121,128]
AQP10b	Seabream	Seabream	Seabream	-	-	[129]
AQP11	Pig	Pig Stallion *	Pig	-	Mouse ^#^	[104,122,130,131,132]
	Bull		Bull			
	Stallion *		Rat			
			Stallion *			

* Immunolocalization data unavailable. ^#^ Only mRNA was identified.
